# A Comprehensive Study of Repetitive Transcranial Magnetic Stimulation in Parkinson's Disease

**DOI:** 10.5402/2011/845453

**Published:** 2011-08-08

**Authors:** Hideki Kimura, Masayuki Kurimura, Katsurou Kurokawa, Utako Nagaoka, Shigeki Arawaka, Manabu Wada, Toru Kawanami, Keiji Kurita, Takeo Kato

**Affiliations:** Department of Neurology, Hematology, Metabolism, Endocrinology, and Diabetology, Faculty of Medicine, Yamagata University, 2-2-2 Iida-nishi, Yamagata 990-9585, Japan

## Abstract

The clinical benefits of repetitive transcranial magnetic stimulation (rTMS) for Parkinson's disease (PD) remain controversial. We performed a comprehensive study to examine whether rTMS is a safe and effective treatment for PD. Twelve PD patients received rTMS once a week. The crossover study design consisted of 4-week sham rTMS followed by 4-week real rTMS. The Unified Parkinson's Disease Rating Scale (UPDRS), Modified Hoehn and Yahr Stage, Schwab and England ADL Scale, Actigraph, Mini-Mental State Examination, Hamilton Depression Scale, Wechsler Adult Intelligence Scale-revised, and cerebral blood flow (CBF) and cerebrospinal fluid (CSF) examinations were used to evaluate the rTMS effects. Under both drug-on and drug-off conditions, the real rTMS improved the UPDRS scores significantly, while the sham rTMS did not. There were no significant changes in the results of the neuropsychological tests, CBF and CSF. rTMS seems to be a safe and effective therapeutic option for PD patients, especially in a wearing-off state.

## 1. Introduction

Parkinson's disease (PD) is the second most common neurodegenerative disease after Alzheimer's disease. The prevalence of PD in Japan has been estimated to be about 100 per 100,000 population [1]. The exact etiology and pathogenesis of PD remain unknown at present [2–5]. The treatments for PD consist of antiparkinsonian drugs, such as L-dopa, and stereotactic brain surgery. Although these treatments are effective for PD symptoms, there are several therapeutic problems, such as the on-and-off phenomenon. Therefore, to overcome the problems, some therapeutic trials for PD are being conducted. Among them, repetitive transcranial magnetic stimulation (rTMS) has been used with some PD patients. In 1994, Pascual-Leone et al. reported that rTMS improved the fine movement of the upper extremities in patients with PD [6]. Since then, clinical trials of rTMS for PD have been reported, many of which indicated the efficacy of rTMS on the symptoms of PD [7–11]; however, others did not [12–14]. Moreover, no study has performed a comprehensive analysis including examinations of neuropsychological status, cerebral blood flow (CBF), and cerebrospinal fluid (CSF). Here, we report a comprehensive clinical trial of rTMS for PD with blind tests of motor functions.

## 2. Patients and Methods

### 2.1. Patients

Twelve PD patients (seven men and five women) with a mean age of 69.2 years (range: 57–78 years) were included in this study (Table 1). We used the diagnostic criteria recommended by the “Multicentric Research Study on the Skill and Indication of Surgical Therapy for PD,” a research group supported by the Japanese government. In brief, PD was defined as the presence of all of the following five items: (1) insidious onset after 20 years of age, (2) resting tremor of 4–6 Hz or cogwheel rigidity with akinesia or small-step gait, (3) apparent improvement of parkinsonism by L-dopa, a dopamine receptor agonist, or an anticholinergic agent, (4) no history of administration of drugs known to cause parkinsonism, and (5) exclusion of symptomatic parkinsonism, such as vascular parkinsonism, multiple system atrophy, progressive supranuclear palsy, or normal-pressure hydrocephalus. All the patients gave informed consent before entering the study. The Ethical Committee of Yamagata University, Faculty of Medicine, approved the study.

### 2.2. Protocol of rTMS Treatment

TMS was performed with an SNM-1100 magnetic stimulator with a YM-121B large round coil (Nihon Koden, Tokyo, Japan). The stimulus parameters and the treatment protocol in the present study were according to the study of Shimamoto and Shigemori [7], which showed a therapeutic effect of TMS on the symptoms of PD. In brief, the stimulus intensity was set at 700 V, and the stimulus frequency was 0.2 Hz. To stimulate the motor and supplemental motor areas, the real TMS was applied in the frontal region with the coil placed horizontally at 3 cm anterior from the vertex to the lateral angle of the eyes. The real TMS induced a motor-evoked potential (MEP) of the muscles of the lower extremities by stimulating the motor area of the cerebral cortex. The sham TMS was applied with the coil placed vertically at 5% anterior from Fz according to the 10–20 system. The sham TMS did not induce any MEP. The real and sham stimulations were applied 30 times in the clockwise direction and 30 times in the counterclockwise direction by the electric current in the coil. The rTMS study design consisted of sham rTMS treatments for the first four weeks followed by real rTMS treatments for the next four weeks. Both the sham and real rTMS treatments were performed once per week. The study design was in accordance with the recommendation for rTMS proposed by the Japanese Society of Clinical Neurophysiology [15].

### 2.3. Estimation of Therapeutic Outcome

For the quantitative estimation of the outcome of rTMS treatments, the following clinical test batteries were used. The motor function and severity of PD were estimated by the Unified Parkinson's Disease Rating Scale (UPDRS), Modified Hoehn and Yahr Stage, Schwab and England ADL Scale, and Actigraph. For the estimation of the psychiatric effects of rTMS, the Mini-Mental State Examination (MMSE), Hamilton Depression Scale (HAM-D), and Wechsler Adult Intelligence Scale-revised (WAIS-R) were used. For the estimation of changes in cerebral blood flow (CBF) and possible harmful effects on the brain, single photon emission computed tomography (SPECT) and cerebrospinal fluid (CSF) examinations were conducted, respectively.

### 2.4. Motor Function and Severity of PD

Board-certified neurologists blindly estimated the motor function and severity of the parkinsonian symptoms. Without having any information of the patients on the drug-on or drug-off and the conditions of real or sham rTMS, the neurologists viewed a video record of each patient under six conditions: before sham rTMS (drug-on and drug-off), after sham rTMS (drug-on and drug-off), and after real rTMS (drug-on and drug-off). Under the drug-on condition, patients took antiparkinsonian drugs as prescribed. Under the drug-off condition, the morning doses of L-dopa compounds, bromocriptine, amantadine, and trihexiphenidyl were aborted on the examination day. The doses of pergolide were stopped from the night of the day before the examination day. The doses of cabergoline and selegiline were stopped from the morning of the day before the examination day. Patients took the prescribed antiparkinsonian drugs immediately after the drug-off examinations. 

UPDRS was scored at six states: before treatment (drug-on and drug-off), after sham rTMS treatment (drug-on and drug-off), and after real rTMS treatment (drug-on and drug-off). The Modified Hoehn and Yahr Stage was used to estimate the severity of PD patients. Stage 0 indicates no symptoms, and Stage 5, the worst state. The Schwab and England ADL Scale is a scale of activity of daily life, and it is scored from 0 to 100% according to the independence of voluntary actions in daily life. The Modified Hoehn and Yahr Stage and Schwab and England ADL Scale were scored under the same six conditions as for UPDRS scoring. 

An Actigraph is a wrist watch-like electronic device that records the frequency of arm movements (Ambulatory Monitoring, Inc., Ardsley, NY, USA) [16, 17]. The device was set to the Zero Crossing Mode, which counts the times of acceleration over 0.1 G. The Actigraph examination was performed under three states: before treatment, after sham rTMS treatment, and after real rTMS treatment. Patients wore the device on the nondominant arm for three days in each state. We analyzed the acceleration count data collected by the Actigraph with “Action W” software (supplied with the device) and obtained the mean counts per minute and the percentage of time of resting (the period that no count was recorded in a minute) while the subjects were awake and asleep.

### 2.5. Psychiatric Effects

The effects on the patients' psychiatric conditions were examined by the MMSE, WAIS-R, and HAM-D. The MMSE is a question battery consisting of orientation, registration of words, attention, calculation, recalling of words, language, and visual construction [18]. Intelligence was also measured by the WAIS-R. Depressive symptoms were estimated using the HAM-D [19]. The MMSE, WAIS-R, and HAM-D were scored under the three conditions: before treatment, after sham rTMS treatment, and after real rTMS treatment.

### 2.6. Measurements of CBF

CBF was estimated by brain SPECT. ^133^Xe was used as a radionuclide. CBF was measured under three conditions: before treatment, after sham rTMS treatment, and after real rTMS treatment.

### 2.7. Examination of CSF

CSF examinations were performed twice: before sham rTMS treatment and after real rTMS treatment. Total protein, monoamine metabolites (homovanillic acid (HVA), 5-hydroxy indoleacetic acid (5-HIAA), 3-methoxy-4-hydroxyphenylethylene glycol (MHPG)), and neuron-specific enolase (NSE) in CSF were measured. The total protein concentration was determined by the pyrogallol-red method. The concentrations of HVA, 5-HIAA, and MHPG were determined by high-performance liquid chromatography (HPLC) [20]. NSE was determined with the radioimmunoassay [21].

### 2.8. Statistical Analysis

Statistical analysis was performed with the SPSS 11.0 statistical analysis package (SPSS Inc., Chicago, Ill, USA). The repeated measures ANOVA (analysis of variance) were used to analyze the results of the UPDRS, Modified Hoehn and Yahr Stage, Schwab and England ADL Scale, Actigraph, MMSE, HAM-D, and CBF examinations. The paired *t*-test with Bonferroni correction was used for post hoc analysis of the results of UPDRS. The one-way ANOVA was used to analyze the results of WAIS-R. The paired *t*-test was used to analyze the results of CSF examinations. Statistical significance was accepted at *P* < 0.05.

## 3. Results

Subjective improvements of resting tremor (five patients), painful dystonia of the legs (one patient), voice loudness (three patients), bending posture (one patient), wearing-off phenomenon (one patient), and akinesia (one patient) were observed after the rTMS treatments, and these lasted for one to three months. 

Under the drug-on conditions, the UPDRS scores before treatment, after sham rTMS, and after real rTMS were 48.45 ± 12.36 (mean ± standard deviation), 43.58 ± 12.33, and 40.15 ± 11.99, respectively (Table 2). Through the three examinations, the UPDRS scores changed significantly (*P* = 0.006, repeated measures ANOVA). The real rTMS treatment improved the UPDRS scores significantly (*P* = 0.029, paired *t*-test with Bonferroni correction), while the sham rTMS treatment did not (*P* = 0.066, paired *t*-test with Bonferroni correction) (Table 2). The Modified Hoehn and Yahr Stages before treatment, after sham rTMS, and after real rTMS were 2.92 ± 0.56, 2.75 ± 0.69, and 2.88 ± 0.57, respectively, and did not show significant changes (*P* = 0.132, repeated measures ANOVA) (Table 2). Schwab and England ADL scales before treatment, after sham rTMS, and after real rTMS were 73.33 ± 14.35, 72.50 ± 15.45, and 75.00 ± 14.46, respectively, and did not show significant changes (*P* = 0.442, repeated measures ANOVA) (Table 2).

Under the drug-off conditions, the UPDRS scores before treatment, after sham rTMS, and after real rTMS were 53.55 ± 19.96, 48.78 ± 14.32, and 44.29 ± 15.40, respectively (Table 2). Through the three examinations, the UPDRS scores under the drug-off conditions changed significantly (*P* = 0.003, repeated measures ANOVA). The real rTMS treatment improved the UPDRS scores significantly (*P* = 0.015, paired *t*-test with Bonferroni correction), while the sham rTMS treatment did not (*P* = 0.454, paired *t*-test with Bonferroni correction) (Table 2). The Modified Hoehn and Yahr Stages before treatment, after sham rTMS, and after real rTMS were 2.92 ± 0.56, 2.92 ± 0.70, and 2.88 ± 0.57, respectively, and did not show significant changes (*P* = 0.590, repeated measures ANOVA) (Table 2). The Schwab and England ADL scales before treatment, after sham rTMS, and after real rTMS were 70.00 ± 17.06, 67.50 ± 18.65, and 73.33 ± 13.71, respectively, and did not show significant changes (*P* = 0.093, repeated measures ANOVA) (Table 2).

The results of the Actigraph examination (mean count per one minute and percentage of time of resting) while the subjects were awake or asleep did not change significantly among the three conditions (before treatment, after sham rTMS, and after real rTMS) (Table 3). The MMSE scores before treatment, after sham rTMS, and after real rTMS were 25.33 ± 3.03, 25.42 ± 2.97, and 26.58 ± 2.97, respectively, and the scores slightly improved through the three trials, but the changes were not significant (*P* = 0.114, repeated measures ANOVA) (Table 3). The HAM-D scores before treatment, after sham rTMS, and after real rTMS were 11.25 ± 6.30, 10.33 ± 4.91, and 9.42 ± 4.32, respectively (Table 3). The scores slightly improved through the sham and real rTMS treatments, but the change was not significant (*P* = 0.447, repeated measures ANOVA) (Table 3). In the WAIS-R test, the intelligence quotients did not change significantly among the three conditions (Table 3). In the CBF examination, no significant changes were observed in the blood flow among the three conditions (Table 3). In the CSF examination, the concentrations of HVA, 5-HIAA, MHPG, or NSE did not significantly change between the two conditions (before rTMS and after real rTMS) (Table 3).

## 4. Discussion

In the present study, the real rTMS treatments significantly improved the scores of UPDRS in PD; however, the sham rTMS treatments did not, indicating that rTMS may be effective for PD symptoms. In the clinical settings, it is a noteworthy finding that the rTMS treatment had a therapeutic effect under the drug-off condition, as it is analogous to the wearing-off phenomenon. In the advanced stage of PD, patients suffer from the instability of drug effects, such as the wearing-off or on-off phenomenon [22]. The present result suggests that the real rTMS treatment may be useful to prevent intensification of symptoms due to the wearing-off phenomenon in patients with PD. 

The scores of Actigraph, Schwab and England ADL Scale, and Modified Hoehn and Yahr Stage were not significantly changed before and after rTMS. Since the degree of improvements of PD symptoms by rTMS was small in the present study, it seems that only the UPDRS was able to detect this small difference of the symptomatic improvement of PD. In other words, for the functional evaluation of PD patients, the UPDRS seems to be the most sensitive among the functional measures used. 

The present study has several advantages over previous studies using rTMS for PD. Each study was different in terms of the coils used and the sites, intensities, frequencies, and total number of stimulations. Accordingly, the results cannot be compared equally. However, the present study had clear methodological advantages over previous studies. Firstly, we applied both the sham and real rTMS treatments in a crossover design. Secondly, the evaluations were performed under both drug-on and drug-off conditions. Thirdly, we estimated motor functions by the UPDRS score in a blind manner. Lastly, we performed a comprehensive study including the neuropsychological, CBF, and CSF examinations as well as the motor function of PD.

In the present study, no adverse effects of the rTMS were observed. The MMSE scores slightly improved through the three trials, but the changes were not statistically significant. In the WAIS-R tests, the verbal IQ and performance IQ showed a slight improvement but without a statistical significance. The possibility cannot be excluded that, although not significant, the slight improvement of the results of the WAIS-R tests may be due to a learning effect. In any case, the results seem to indicate that the rTMS treatment may not have any significant beneficial or adverse effect on mental and psychiatric conditions. In addition, the rTMS treatment in our protocol did not appear to cause any damage in the central nervous system of the patients because the CBF and CSF concentrations of total protein and NSE did not change before and after rTMS. NSE is known to increase in CSF when neurons are injured rapidly, as in Creutzfeldt-Jakob disease and in the acute stage of cerebral infarction [23, 24]. 

The mechanism of the therapeutic effect of rTMS for PD has not been clarified. Dopaminergic antiparkinsonian drugs have been reported to increase the CSF concentration of the monoamine metabolites, including HVA [25]. In the present study, the motor symptoms were significantly improved in the PD patients examined, but the CSF concentrations of HVA, 5-HIAA, and MHPG did not increase. The results suggest that the improvement of PD symptoms by rTMS may not be mediated by an increase in the monoamine concentration. Furthermore, the rTMS treatment did not increase CBF in the present study, indicating that the therapeutic effects of the rTMS in the protocol may not be mediated by an increase in CBF. 

One possible explanation for the therapeutic effects of the rTMS on PD symptoms is as follows. In PD, the excitability of the cerebral cortex is suspected to be decreased because of the altered excitatory inputs from the thalamus; TMS may compensate for this decreased excitability of the cerebral cortex [26–28]. Another explanation is that TMS may improve the imbalance between the substantia nigra pars reticulata and the internal segment of the globus pallidus. The feedback system from the cerebral cortex to the striatum may be stimulated with rTMS, resulting in the normalization of the imbalance. Further studies are needed to elucidate the exact therapeutic mechanism by rTMS.

In conclusion, the present study demonstrates that rTMS seems to be a safe and effective therapeutic option for PD symptoms, especially in the wearing-off state.

## Figures and Tables

**Table 1:** Profiles of 12 PD patients.

Case	Sex	Age (years)	Disease duration (years)	Modified Hoehn and Yah Stage (1)	Schwab and England ADLScale (1)	L-DOPA (2) (mg)	D2 agonists (3)		Amantadine (mg)	Droxydopa (mg)	Trihexi-phenidyl (mg)	Other drugs
							Drugs	Dosage (mg)				
1	Man	57	1	3	80%	300*	CB	1	0	0	0	Maprotiline, flunitrazepam
2	Man	67	13	3	60%	300	BC	7.5	150	300	4	
3	Woman	70	8	2.5	80%	300	none	—	0	300	0	
4	Man	66	6	2.5	80%	300	PG	0.3	100	600	4	
5	Man	64	9	2.5	80%	400	BC	15	0	200	4	
6	Woman	76	14	3	70%	500	BC	10	50	0	0	Imipramine, flurazepam
7	Man	73	11	4	50%	400	PG	1	0	900	0	Imipramine, clonazepam
8	Woman	69	2	3	80%	300	PG	0.1	0	600	0	
9	Woman	75	12	2.5	60%	300	PG	0.75	100	0	0	
10	Man	78	12	4	30%	400	PG	0.75	50	600	0	Mitodrine
11	Man	67	4	2.5	90%	450	PG	0.75	0	0	6	
12	Woman	68	10	2	80%	450	BC	15	0	0	6	

(1) Under the drug-off state at before sham rTMS.

(2) Levodopa-carbidopa combination. *Levodopa-benserazide combination.

(3) CB: cabergolin, BC: bromocriptine, PG: pergolide.

**Table:** (a)

		Before rTMS	After sham rTMS	After real rTMS	*P* value
Drug-on	UPDRS	48.45 ± 12.36	43.58 ± 12.33	40.15 ± 11.99	0.006*
	m H & Y	2.92 ± 0.56	2.75 ± 0.69	2.88 ± 0.57	0.132
	S&E	73.33 ± 14.35	72.50 ± 15.45	75.00 ± 14.46	0.442

Drug-off	UPDRS	53.55 ± 19.96	48.78 ± 14.32	44.29 ± 15.40	0.003*
	m H & Y	2.92 ± 0.56	2.92 ± 0.70	2.88 ± 0.57	0.590
	S&E	70.00 ± 17.06	67.50 ± 18.65	73.33 ± 13.71	0.093

Repeated measures ANOVA. *P < 0.05.

**Table:** (b)

		Before rTMS	After sham rTMS	After real rTMS	Sham (1)	Real (2)	Overall (3)
Drug-on	UPDRS	48.45 ± 12.36	43.58 ± 12.33	40.15 ± 11.99	0.066	0.029*	0.005*
Drug-off	UPDRS	53.55 ± 19.96	48.78 ± 14.32	44.29 ± 15.40	0.454	0.015*	0.026*

Paired *t*-test with Bonferroni correction. *P < 0.05. Drug-on: taking drugs as prescribed, drug-off: according to Table 2. UPDRS: unified Parkinson's Disease Rating Scale. S&E: Schwab and England ADL scale, mH & Y: modified Hoehn and Yahr stage. (1): *P* value for between before rTMS and after sham rTMS. (2): *P* value for between after sham rTMS and after real rTMS. (3): *P* value for between before rTMS and after real rTMS.

**Table 3:** Changes of Actigraph, MMSE, HAM-D, WAIS-R, cerebral blood flow, and cerebrospinal fluid by rTMS (sham and real).

		Before rTMS	After sham rTMS	After real rTMS	*P* value
Actigraph up state (1)	Mean counts per one minute	131.2 ± 17.0	131.4 ± 27.2	130.1 ± 31.7	0.977
	Resting (%)	17.1 ± 4.5	18.9 ± 7.2	19.0 ± 8.6	0.611
Actigraph down state (2)	Mean counts per one minute	31.0 ± 24.4	31.0 ± 19.6	35.2 ± 26.8	0.652
	Resting (%)	80.8 ± 17.5	76.7 ± 15.2	75.9 ± 15.9	0.593

MMSE		25.33 ± 3.03	25.42 ± 2.97	26.58 ± 2.97	0.114
HAM-D		11.25 ± 6.30	10.33 ± 4.91	9.42 ± 4.32	0.447

WAIS-R^#^	Verbal IQ	97.30 ± 20.63	101.63 ± 18.62	98.80 ± 20.80	0.901
	Performance IQ	89.60 ± 10.45	96.00 ± 9.41	99.80 ± 9.36	0.159
	Total IQ	93.40 ± 16.78	99.00 ± 14.68	98.00 ± 16.93	0.742

Cerebral blood flow	(ml/min/100 g)	47.50 ± 3.26	45.75 ± 3.55	47.75 ± 3.96	0.078

Cerebrospinal fluid^##^	Total protein (mg/dl)	41.2 ± 13.1		39.9 ± 14.7	0.579
	HVA (ng/ml)	35.3 ± 17.9		36.2 ± 12.8	0.817
	5-HIAA (ng/ml)	13.4 ± 7.7		13.7 ± 7.6	0.799
	MHPG (ng/ml)	9.5 ± 2.9		9.0 ± 2.5	0.368
	NSE (ng/ml)	15.5 ± 9.2		15.4 ± 7.6	0.952

Repeated measures ANOVA, *P < 0.05. ^#^One-way ANOVA. ^##^Paired *t*-test. (1): Actigraph record while awaken. (2): Actigraph record while sleeping. MMSE: Mini-Mental State Examination, HAM-D: Hamilton Depression Scale. WAIS-R: Wechsler Adult Intelligence Scale-Revised, IQ: intellectual quotient. HVA: homovanillic acid; 5-HIAA: 5-hydroxy indoleacetic acid; MHPG: 3-methoxy-4-hydroxyphenylethylene glycol. NSE: neuron-specific enolase.

## References

[1] Kimura H, Kurimura M, Wada M (2002). Female preponderance of Parkinson’s disease in Japan. *Neuroepidemiology*.

[2] Arawaka S, Wada M, Goto S (2006). The role of G-protein-coupled receptor kinase 5 in pathogenesis of sporadic Parkinson’s disease. *Journal of Neuroscience*.

[3] Karube H, Sakamoto M, Arawaka S (2008). N-terminal region of *α*-synuclein is essential for the fatty acid-induced oligomerization of the molecules. *FEBS Letters*.

[4] Machiya Y, Hara S, Arawaka S (2010). Phosphorylated *α*-synuclein at Ser-129 is targeted to the proteasome pathway in a ubiquitin-independent manner. *Journal of Biological Chemistry*.

[5] Arawak S, Machiya Y, Kato T (2010). Heat shock proteins as suppressors of accumulation of toxic prefibrillar intermediates and misfolded proteins in neurodegenerative diseases. *Current Pharmaceutical Biotechnology*.

[6] Pascual-Leone A, Valls-Solé J, Brasil-Neto JP, Cammarota A, Grafman J, Hallett M (1994). Akinesia in Parkinson’s disease. II. Effects of subthreshold repetitive transcranial motor cortex stimulation. *Neurology*.

[7] Shimamoto H, Shigemori M (1999). Therapeutic effect of repetitive transcranial magnetic stimulation. *Shinkei Naika*.

[8] Mally J, Stone TW (1999). Improvement in Parkinsonian symptoms after repetitive transcranial magnetic stimulation. *Journal of the Neurological Sciences*.

[9] Siebner HR, Mentschel C, Auer C, Conrad B (1999). Repetitive transcranial magnetic stimulation has a beneficial effect on bradykinesia in Parkinson’s disease. *NeuroReport*.

[10] Fukudome T, Goto H, Izumoto H, Matsuo H, Shibuya N (2002). The effects of repetitive transcranial magnetic stimulation (rTMS) in the patients with Parkinson’s disease. *Rinsho Shinkeigaku*.

[11] Málly J, Farkas R, Tóthfalusi L, Stone TW (2004). Long-term follow-up study with repetitive transcranial magnetic stimulation (rTMS) in Parkinson’s disease. *Brain Research Bulletin*.

[12] Ghabra MB, Hallett M, Wassermann EM (1999). Simultaneous repetitive transcranial magnetic stimulation does not speed fine movement in PD. *Neurology*.

[13] Tergau F, Wassermann EM, Paulus W, Ziemann U (1999). Lack of clinical improvement in patients with Parkinson’s disease after low and high frequency repetitive transcranial magnetic stimulation. *Electroencephalography and Clinical Neurophysiology. Supplement*.

[14] Okabe S, Ugawa Y, Kanazawa I (2003). 0.2-Hz repetitive transcranial magnetic stimulation has no add-on effects as compared to a realistic sham stimulation in parkinson’s disease. *Movement Disorders*.

[15] Kimura J, Mano Y, Ugawa Y (1999). Proposals for safety and clinical application of high frequency transcranial magnetic stimulation. *Nouha to Kindenzu*.

[16] Kurita K, Wada M, Kurimura M, Kawanami T, Kato T (1999). An actigraphy study on the activity of caregivers for patients with neurodegenerative diseases. *Annual Report of the Research Committee of Medico-Welfare Network Construction for Supporting Severely Disabled Patients with Specific Diseases*.

[17] Monk TH, Buysse DJ, Rose LR (1999). Wrist actigraphic measures of sleep in space. *Sleep*.

[18] Tombaugh TN, McIntyre NJ (1992). The mini-mental state examination: a comprehensive review. *Journal of the American Geriatrics Society*.

[19] Rosenberg R (2000). Outcome measures of antidepressive therapy. *Acta Psychiatrica Scandinavica. Supplementum*.

[20] Scheinin M, Chang WH, Kirk KL, Linnoila M (1983). Simultaneous determination of 3-methoxy-4-hydroxyphenylglycol, 5-hydroxyindoleacetic acid and homovanillic acid in cerebrospinal fluid with high-performance liquid chromatography using electrochemical detection. *Analytical Biochemistry*.

[21] Vermuyten K, Lowenthal A, Karcher D (1990). Detection of neuron specific enolase concentrations in cerebrospinal fluid from patients with neurological disorders by means of a sensitive enzyme immunoassay. *Clinica Chimica Acta*.

[22] Souza RG, Borges V, Silva SMCDA, Ferraz HB (2007). Quality of life scale in Parkinson’s disease: PDQ-39—(Brazilian Portuguese version) to assess patients with and without levodopa motor fluctuation. *Arquivos de Neuro-Psiquiatria*.

[23] Zerr I, Bodemer M, Räcker S (1995). Cerebrospinal fluid concentration of neuron-specific enolase in diagnosis of Creutzfeldt-Jakob disease. *The Lancet*.

[24] Hay E, Royds JA, Davies-Jones GAB (1984). Cerebrospinal fluid enolase in stroke. *Journal of Neurology Neurosurgery and Psychiatry*.

[25] Gumpert J, Sharpe D, Curzon G (1973). Amine metabolites in the cerebrospinal fluid in Parkinson’s disease and the response to levodopa. *Journal of the Neurological Sciences*.

[26] Alexander GE, DeLong MR, Strick PL (1986). Parallel organization of functionally segregated circuits linking basal ganglia and cortex. *Annual Review of Neuroscience*.

[27] Alexander GE, Crutcher MD (1990). Functional architecture of basal ganglia circuits: neural substrates of parallel processing. *Trends in Neurosciences*.

[28] DeLong MR (1990). Primate models of movement disorders of basal ganglia origin. *Trends in Neurosciences*.

